# A systematic review of the psychometric properties of the cross-cultural translations and adaptations of the Multidimensional Perceived Social Support Scale (MSPSS)

**DOI:** 10.1186/s12955-018-0912-0

**Published:** 2018-05-02

**Authors:** Jermaine M. Dambi, Lieselotte Corten, Matthew Chiwaridzo, Helen Jack, Tecla Mlambo, Jennifer Jelsma

**Affiliations:** 1 Division of Physiotherapy, School of Health and Rehabilitation Sciences, University of Cape Town, Observatory, Cape Town, South Africa; 2 Rehabilitation Department, College of Health Sciences, University of Zimbabwe, P.O Box A178, Avondale, Harare, Zimbabwe; 3 King’s College London, Institute of Psychiatry, Psychology, and Neuroscience, London, England; 4 Harvard Medical School, Boston, MA USA

**Keywords:** Multidimensional perceived social support, Translation, Adaptation, Validation, Reliability, Validity

## Abstract

**Background:**

Social support (SS) has been identified as an essential buffer to stressful life events. Consequently, there has been a surge in the evaluation of SS as a wellbeing indicator. The Multidimensional Perceived Social Support Scale (MSPSS) has evolved as one of the most extensively translated and validated social support outcome measures. Due to linguistic and cultural differences, there is need to test the psychometrics of the adapted versions. However, there is a paucity of systematic evidence of the psychometrics of adapted and translated versions of the MSPSS across settings.

**Objectives:**

To understand the psychometric properties of the MSPSS for non-English speaking populations by conducting a systematic review of studies that examine the psychometric properties of non-English versions of the MSPSS.

**Methods:**

We searched Africa-Wide Information, CINAHL, Medline and PsycINFO, for articles published in English on the translation and or validation of the MSPSS. Methodological quality and quality of psychometric properties of the retrieved translations were assessed using the COSMIN checklist and a validated quality assessment criterion, respectively. The two assessments were combined to produce the best level of evidence per language/translation.

**Results:**

Seventy articles evaluating the MSPSS in 22 languages were retrieved. Most translations [16/22] were not rigorously translated (only solitary backward-forward translations were performed, reconciliation was poorly described, or were not pretested). There was poor evidence for structural validity, as confirmatory factor analysis was performed in only nine studies. Internal consistency was reported in all studies. Most attained a Cronbach’s alpha of at least 0.70 against a backdrop of fair methodological quality. There was poor evidence for construct validity.

**Conclusion:**

There is limited evidence supporting the psychometric robustness of the translated versions of the MSPSS, and given the variability, the individual psychometrics of a translation must be considered prior to use. Responsiveness, measurement error and cut-off values should also be assessed to increase the clinical utility and psychometric robustness of the translated versions of the MSPSS.

**Trial registration:**

PROSPERO - CRD42016052394.

**Electronic supplementary material:**

The online version of this article (10.1186/s12955-018-0912-0) contains supplementary material, which is available to authorized users.

## Background

Social support (SS) is an essential buffer to stressful life events [1–13]. An adequate amount of SS improves mental health by mitigating the effects of negative psychosocial outcomes such as depression, anxiety, low self-efficacy, stress and loneliness or social isolation [1, 3, 4, 6, 9, 14–16]. Further, SS is a multidimensional, latent variable that dependents upon an individual’s politico-social environment, socialization process and personal values/ethos amongst other factors [1, 3, 10–12, 17, 18]. The conceptualization and perception of SS is both complex and diverse, as testified by a plethora of conceptual frameworks and definitions which have been postulated to describe this subjective and yet important phenomenon [12, 13, 15, 16]. Social support can be defined as the amount of assistance one gets through interactions with other people [15, 16]. The support can be either emotional (e.g. empathy), tangible (e.g. practical help) or informational (e.g. advice) [15, 16, 19, 20].

Various outcome measures have been developed to measure SS [3, 11]. Originally created to measure SS in American adolescents, the Multidimensional Scale of Perceived Social Support (MSPSS) has evolved as one of the most extensively used SS outcome measure [3, 6–8, 11, 12, 18]. The MSPSS has 12 items that measure the perceived adequacy of the available amount of SS [15, 21, 22] (See Additional file 1). It measures the amount of SS an individual receives from three sources i.e. friends, family and significant other/special person. The amount of SS is rated on a seven-point Likert scale; with responses ranging from very strongly disagree (=1) to very strongly agree (=7). The cumulative/total scores ranges from 12 to 84. As no item response theory calibration has been applied to the tool, the scores are interpreted as, the higher the score, the greater the amount of available SS [21]. The original version of the MSPSS yielded a three-factor structure, high internal consistency (α = 0.88), stability (yielded α = 0.85 after 3 months from first administration) and moderate construct validity as the SS scores were negatively correlated to anxiety (*r* = − 0.18; *p* < 0.01) and depression scores (*r* = − 24; *p* < 0.01) [22].

The drive towards evidence-based practise (EBP) and patient-centred care has led to an increase in the cross-cultural adaptation and translation of patient-reported outcome measures (PROMs) [7, 23–25]. To this end, the MSPSS has undergone extensive translation and adaption across linguistic and socio-economic contexts and settings i.e. from low- [7, 13, 26] and middle [11, 18, 27–29] to high-income countries [3, 6, 9, 17, 18, 30–34]. However, evidence of the psychometric properties of the translated and adapted versions of the MSPSS is fragmented, but deeply important to both clinical practice and research. For instance, due to conceptual differences, some authors have collapsed the scoring system (response options) to three [30, 35], four [12], five [13, 26, 36], and six [31] levels against the original seven-point Likert scoring system. However, the category “reordering” was based on “qualitative” analysis yet in those circumstances, item response theory techniques such as Rasch analysis are a prerequisite for ensuring the interpretability of response categories for translated tools [37, 38]. Secondly, due to linguistic differences, some of the terminology of the original MSPSS have been changed, for example, the term “someone special” has been replaced by the term “husband” for some translations [5]. Thirdly, cultural differences are likely to influence perception of social support [10–12, 17, 18] thus potentially influencing the structural validity of the translated versions of the MSPSS. For example, single factor structures were reproduced in the validation of the MSPSS in Asian countries such as Turkey [39–42], Thailand [28, 43, 44] and Pakistan [45–49], which are considered as “collectivistic” societies [50]. This implies that respondents could not differentiate between support provided by family, friends and significant others as postulated by the developers. The MSPSS was originally validated in the US which is considered an “individualistic” society [51]. Given the wide variation in MSPSS translations and cultural adaptations, a systematic evaluation of the adapted and translated versions of the MSPSS will help bring an understanding of the quality of the existing tools, and gaps in knowledge and adaptation [7, 23, 24, 52]. Further, a recent literature review asserts the psychometric robustness of the MSPSS across various settings and study populations [53]. However, the methodologies of the cited studies were not critically appraised. This is a limitation as the reported psychometrics are dependent on the quality of the methodologies applied in accordance with the COnsensus-based Standards for the selection of health status Measurement Instruments (COSMIN) criterion [54–57] . For instance, issues such as the sampling, handling of missing responses, the model used for analysis, bias in research reporting amongst other factors affects both the internal and external validity of the psychometric evaluation studies [55, 56, 58]. More so, other psychometric properties such as floor and ceiling effects, critical/cut off values and responsiveness were not reported and this weakens the level of evidence of the purported psychometric robustness of the MSPSS [53]. Therefore, the aims of the present review are to: (I) systematically identify the translated and adapted versions of the MSPSS; (II) evaluate the methodologies applied in the adaptation and translation process; and (III) appraise the psychometric properties of the translated and adapted versions.

## Methods

### Protocol and registration

This review was conducted in accordance with the Preferred Reporting Items of Systematic Reviews and Meta-Analyses Protocol (PRISMA) guidelines (Additional file 2). The protocol was registered with and published on the PROSPERO database (Ref-CRD42016052394) [59].

### Eligibility criteria

Studies were included if one of the aim(s) was to: translate and culturally adapt the MSPSS; evaluate the psychometrical properties of translated version(s) of the MSPSS or if they measured SS using translated versions of the MSPSS. Additionally, only studies applying a quantitative study designs and reporting on the psychometrics of the translated, 12-item version of the MSPSS were included. Due to limitation in resources for translation, only full text articles published in English were included. Studies based in more than one country that applied the MSPSS as an outcome measure, commentaries, review articles and studies which utilized several linguistic versions of the MSPSS were excluded. Additionally, studies which utilized both the original version of the MSPSS and the translated version were also excluded as the aim of the review was to appraise the psychometric properties of the translated versions of the MSPSS.

### Information sources

We searched Africa-Wide information, CINAHL, PubMed, Psych INFO, and Scopus for peer-reviewed articles. Google Scholar was also searched to identify grey literature such as dissertations. We also contacted the developer of the MSPSS to identify the translations which we might have missed through database searches. We did not impose a time limit to publication dates to gather as many articles as possible. In cases where the abstract was available online and where it was unclear as to whether the translated version of the MSPSS was applied, the authors were contacted for clarification. Reference lists of the identified articles were manually searched for possible articles for inclusion for the attainment of literature saturation. Literature searches were conducted between November 2016 and February 2017.

### Search

Outlined in Table 1 below is the search strategy applied in retrieving articles on EBSCO-host search engine:

**Table 1:** Search strategy

Key term	Alternative term(s)
multidimensional scale of perceived social support	MSPSS, mspss
translation	translated, translat*
adaption	Cultural adaptation, adapt*
• Language in which the MSPSS was translated to e.g. French	
• Country where the translation was done e.g. France	

As an illustration, articles on the translation and adaptation of the French version of the MSPSS were retrieved as follows: {multidimensional scale of perceived social support OR MSPSS OR mspss} AND {translation OR translated OR translat*} AND {france OR France OR French OR french OR francias}.

### Study selection

A previously described study selection process was utilized [60, 61]. One author (JD) ran the search strategy across all databases. Two independent reviewers (MC and LC) then selected the eligible titles and abstracts for further investigation using a predefined search strategy. Reviewers resolved disagreements about inclusion through discussion, and a, third reviewer (JD) was brought in if the two reviewers could not agree. Another author (MC) manually searched the reference lists of identified articles to screen full texts for inclusion.

### Data collection process

The principal investigator (JD) developed the data collection sheet. The tool/checklist was validated by three co-investigators (MC, TM & JD) with prior experience with psychometrics and psychometrics systematic reviews. The tool was then piloted on ten randomly selected studies of definite rating. Three reviewers (MC, JD & TM) independently applied the COSMIN checklist to rate the methodological quality of the ten studies. The inter-rater reliability was 0.8 as measured by the Kappa coefficient. Most of the disparities emanated from the rating of the structural validity and differences were discussed upon further reference to the COSMIN user manual. Afterwards, two reviewers (MC & TM) independently extracted data from the retrieved studies and their inter-rater reliability was 0.9. The two data collection sheets were reconciled into one data set through discussions between the principal author (JD) and two reviewers (MC & TM).

### Data items

The extracted information included the research setting and design, study sample, demographic and or clinical characteristics of the participants, target translation language and secondary outcome measures e.g. depression. The methodological quality of the translation process and evidence for reliability and validity of the questionnaires was also documented.

### Risk of bias in individual studies

The methodological quality of the retrieved articles was assessed using the COnsensus-based Standards for the selection of health status Measurement Instruments (COSMIN) checklist [54]. It consists of eight boxes which evaluate the methodological quality of the following psychometrics: internal consistency (box A), reliability (box B), measurement error (box C), content validity (box D), structural validity (box E), hypotheses testing (box F), cross-cultural validity (box G), and criterion validity (box H) [54, 55]. Methodological quality is rated on a four-point Likert scale i.e. poor, fair, good and excellent. In assessing a domain, the lowest rating of an item is assigned as the overall quality of the domain under investigation. For example, in assessing structural validity, if an inappropriate rotation method is employed i.e. if orthogonal rotation instead of oblique rotation is used to interpret factors on the MSPSS, the evidence will be rated as poor even if all the other sub-domains are rated as of excellent quality [54–57].

### Best evidence synthesis

To come up with best evidence synthesis for each psychometric property, we used the Cochrane Collaboration Back Review Group criterion [62] as outlined in Table 2 to synthesise results from the COSMIN methodological assessment [54–57] (see Table 3) and Terwee et al. criterion for evaluation of quality of psychometrics [58] (see Table 4).

**Table 2:** Best evidence synthesis of the psychometric properties

Version -Country	Internal consistency	Criterion validity	Construct validity – convergent	Construct validity- divergent	Reproducibility- agreement	Reproducibility- reliability
Arabic women (MSPSS-AW)- USA	Moderate (−-)		Limited (−)	Limited (−)		
Arabic Generic – Lebanon	Limited (−)		Unknown (?)	Unknown (?)		
Chichewa- Malawi	Strong (+++)			Limited (−)		
Chinese (Simplified) – Malaysia	Strong (+++)	Unknown (?)		Unknown (?)		Unknown (?)
Chinese (Traditional) – Hong Kong, China	Conflicting (−)	Unknown (?)	Moderate (−-)	Moderate (−-)		Limited (+)
Chiyao - Malawi	Strong (+++)			Limited (−)		
Creole- USA (Haiti)	Unknown (?)		Unknown (?)			Unknown (?)
French - France	Limited (+)			Limited (−)		Limited (−)
Hausa – Nigeria	Strong (+++)			Limited (−)		Limited (−)
Korean-Korea	Strong (+++)		Limited (−)	Limited (−)		
Luganda - Uganda	Limited (−)					
Malay – Malaysia	Unknown (?)	Unknown (?)		Unknown (?)		Unknown (?)
Persian – Iran	Limited (−)		Unknown (?)			Limited (−)
Polish – Poland	Strong (+++)		Moderate (−-)	Moderate (−-)		
Portuguese – Portugal	Strong (+++)		Limited (−)	Limited (+)	Limited (−)	
Spanish – *USA, ** Spain	Limited (+)	Moderate (−-)	Moderate (−-)			
Swedish – Sweden	Moderate (++)		Limited (−)			Limited (+)
Tamil – Malaysia	Limited (−)	Unknown (?)	Unknown (?)	Unknown (?)		
Thai – Thailand	Moderate (++)		Unknown (?)			Limited (+)
Turkish (Original) - Turkey	Moderate (++)		Conflicting (−)	Conflicting (+)		
Turkish (Revised) – Turkey	Conflicting (−)		Unknown (?)	Unknown (?)		Unknown (?)
Urdu – Pakistan	? (unknown)		Limited (−)	Moderate (−-)		

* and ** denotes findings from the USA and Spain respectively

**Table 3:** Methodological ratings of retrieved studies

Version -Country	Crosscultural validity	Structural validity	Internal consistency	Reliability	Hypothesis testing/construct validity		Criterion validity
					Divergent validity	Convergent validity	
Arabic women – USA	Poor [5]	Fair [5]	Fair [5]	Fair	[5, 47]	Fair [5]	
Arabic Generic – Lebanon	Poor [27]	Poor [27]	Fair [27]	Fair [27]	Fair [27]		
Chichewa- Malawi	Fair [26]	Excellent [26]	Excellent [26]	Fair [26, 36]]			
Chinese (Simplified) – Malaysia	Poor [12]	Poor [12]	Fair [12]	Poor [12]	Poor [12]		Poor [12]
Chinese (Traditional) – Hong Kong, China	Poor [17]	Poor [17] Fair [64, 65]	Excellent [17] Fair [64, 80]	Fair [64]	Poor [17], [73, 76] Fair [64, 75–78, 80–84]	Poor [73, 84] Fair [74, 76, 78–82, 84]	Fair [17]
Chiyao – Malawi	Fair [26]	Excellent [26]	Excellent [26]	Fair [26]			
Creole- Haiti(USA)	Poor [6]		Poor [6]	Fair [6]	Fair [6]		
French – France	Poor [9]	Fair [9]	Fair [9]	Fair [9]	Fair [9]		
Hausa – Nigeria	Good [7, 14]	Excellent [14]	Excellent [14]	Fair [14]	Fair [85]		
Korean-Korea	Poor [87]	Poor [87]	Excellent [87]	Fair [87]	Fair [87]		
Luganda – Uganda	Fair [13]	Poor [13]	Fair [13]				
Malay – Malaysia	Poor [11]	Poor [11, 88]	Fair [11, 88]	Poor [11]	Poor [11, 88]: Fair [89]	Poor [11]	Poor [11]
Persian – Iran	Poor [18]	Poor [18]	Fair [18]	Fair [18]	Poor [90, 91]		
Polish – Poland	Good [92]	Excellent [92]	Excellent [92]		Fair [92, 93], Good [94]	Fair [79, 92, 93], Good [94]	
Portuguese – Portugal	Fair [32]	Excellent [32]	Excellent [32]	Fair [32]	Fair [96, 97], Good [32]	Fair [96]	
Spanish – *USA, ** Spain	Poor ** [99]	Poor * [101], Fair ** [99]	Fair * [101]		Fair ** [34, 98, 99]	Fair ** [34, 98, 99]	
Swedish – Sweden	Good [3]	Poor [3]	Good [3]	Fair [3]		Good [3], Fair [102]	
Tamil – Malaysia	Poor [87]	Poor [87]	Fair [87]		Poor [87]	Poor [87]	Poor [87]
Thai – Thailand	Poor [44]	Good [43, 44]	Good [43, 44]	Fair [44]	Fair [28, 43, 44]	Fair [28, 43, 44]	
Turkish (Original)– Turkey	Poor [39]	Poor [39, 41]	Fair [39, 41]		Poor [41, 42], Fair [39, 40]	Poor [41, 42] Fair [40]	
Turkish (Revised) – Turkey		Fair [29, 104]	Fair [29]	Poor [29]	Fair [29]	Poor [29]	
Urdu – Pakistan	Poor [49]	Poor [49] Fair [47]	Poor [49]		Fair [45–48]	Fair [47]	

* and ** denotes findings from the USA and Spain respectively

**Table 4:** Ratings of quality of psychometric properties

Version –Country	Cross-cultural validity	Structural validity	Internal consistency	Reliability	Construct validity	Criterion validity
Arabic women - USA	? [5]	- [5]	+ [5]		? [63]	
Arabic Generic – Lebanon	? [27]	? [27]	+ [27]		? [27]	
Chichewa- Malawi	- [26]	+ [26]	+ [26]		? [26, 36]	
Chinese (Simplified) – Malaysia	? [12]	? [12]	+ [12]	? [12]	? [12]	? [12]
Chinese (Traditional) – Hong Kong, China	? [17]	? [17], − [64, 80]	? [17], + [64],- [80]	? [17], + [64]	? [17, 64–84]	? [17]
Chiyao – Malawi	- [26]	+ [26]	+ [26]		? [26, 36]	
Creole- Haiti(USA)	? [6]	? [6]	? [6]	? [6]		
French – France	? [9]	- [9]	+ [9]	? [9]	? [9]	
Hausa – Nigeria	- [7, 14]	+ [14]	+ [14]	? [14]	? [85]	
Korean-Korea	? [87]	? [87]	+ [87]		? [87]	
Luganda – Uganda	- [13]	? [13]	? [13]			
Malay – Malaysia	? [11]	? [11, 88]	- [11, 88]	? [11]	? [11, 88], − [89]	? [11]
Persian – Iran	? [18]	? [18]	? [18]	? [18]	? [90, 91]	
Polish – Poland	- [92]	+ [92]	+ [92]		? [92–94]	
Portuguese – Portugal	- [32]	+ [32]	+ [32]	? [32]	? [32, 96, 97]	
Spanish – *USA, ** Spain	? [99] **	? [85] *, − [99] **	+ [85] *, + [99] **		? [34, 84, 99]**, + [98]	
Swedish – Sweden	- [3]	? [3]	+ [3]	+ [3]	+ [3]? [102]	
Tamil – Malaysia	? [103]	? [103]	? [103]		? [103]	? [103]
Thai – Thailand	? [44]	- [43, 44]	+ [43, 44]		? [28, 43, 44]	
Turkish (Original)- Turkey	? [39]	? [39, 41]	+ [39, 41]		? [39–42]	
Turkish (Revised) – Turkey		- [29, 104]	+ [29];? [104]		? [29]	
Urdu – Pakistan	? [49]	? [49], − [47]	? [49]		? [45–48]	

* and ** denotes findings from the USA and Spain respectively

## Results

### Study selection

#### Study characteristics

##### Description of study participants and settings

A total of 22 translations were retrieved from 70 studies. A sample of convenience was the most common method of participant selection [*n* = 7, 31.8%], and translations were most often validated using a cross sectional study design [72.7%, *n* = 16]. Most studies were from high-income settings [72.7%, *n* = 16] and conducted in clinical settings [45.8%, *n* = 11] or at universities [29.1%, *n* = 7]. Participants were of varying ages with the youngest and eldest groups averaging 14.8 (SD 1.6) and 58.7 (SD 13.2) years respectively Table 5.

**Table 5:** Study descriptions

Authors [Year of publication]	Language (s)	Study aim(s)	Design	Country – [income bracket]	Setting	Participants	Sampling	Age in years- Mean [SD]
Aroian et al. [2010]	Arabic	T & PT	Cross-sectional	USA - HIC	Community	Arab Muslim immigrant married woman, *N* = 539	Convenience	40.2 (6.5)
Norries et al. [2011]	Arabic	PT	Cross-sectional	USA - HIC	Community	Arab Muslim immigrant women, *N* = 519	Convenience	40.22 (6.5)
Merhi & Kazarian [2012]	Arabic	T & PT	Cross-sectional	Lebanon - UMIC	Community	Healthy adults, *N* = 221	Not stated	34.0 (11.7)
Stewart et al. [2014]	Chichewa & Chiyao	T & PT	Cross-sectional	Malawi – LIC	Clinical	Women attending antenatal visits, *N* = 583	Convenience	25.1 (6.2)
Stewart et al. [2014]	Chichewa & Chiyao	PT	Cross-sectional	Malawi – LIC	Clinical	Women attending antenatal visits, *N* = 583	Consecutive	25.14 (6.22)
Cao et al. [2015]	Chinese	PT	Cross sectional	China - HIC	Community	Elderly population, *N* = 928	Two-stage stratified cluster sampling	*60–94 [Range]
Chan et al. [2010]	Chinese	PT	Random Controlled Trial	China - HIC	Clinical	Patients with chronic obstructive pulmonary disease, *N* = 206	Random	72.9 (7.7)
He et al. [2016]	Chinese	PT	Cross sectional	China - HIC	Clinical	Burns patients, *N* = 246	Not stated	25.77 (2.14)
Liu et al. [2015]	Chinese	PT	Cross sectional	China - HIC	Community	Adults, *N* = 1471	Not stated	34.5 (10.4)
Meng-Yao et al. [2016]	Chinese	PT	Cross sectional	China - HIC	Clinical	Patients with bladder cancer, *N* = 365	Convenience	63.76 (11.45)
Tan et al. [2016]	Chinese	PT	Cross sectional	China - HIC	High schools	Adolescents, *N* = 618	Random	16.29 (2.58)
Taylor-Piliae et al. [2005]	Chinese	PT	Quasi-experimental	USA-HIC	Community	Chinese nationals with cardio vascular disease risk factors, *N* = 38	Convenience	66 (8.3)
Wang et al. [2014]	Chinese	PT	Cross sectional	China - HIC	Clinical	Patients with depression, *N* = 100	Not stated	41.36 (15.55)
Wang et al. [2015]	Chinese	PT	Longitudinal	China - HIC	Clinical	Patients with breast cancer, *N* = 404	Not stated	47.64 (7.66)
Zeng et al. [2016]	Chinese	PT	Longitudinal	China - HIC	Clinical	Patients mild traumatic brain injury, *N* = 219	Convenience	34.7 (14.8)
Zhang et at [2016]	Chinese	PT	Random Controlled Trial	China - HIC	Clinical	Outpatients with mild depression, *N* = 62	Random	48.3 (17.5)
Zhou et al. [2015]	Chinese	PT	Cross-sectional	China - HIC	Clinical	Patients on methadone maintenance treatment, *N* = 1212	Not stated	42.5 (6.2)
Zhu, Hu & Efird [2012]	Chinese	PT	Cross-sectional, correlational	China - HIC	Community	Elderly population, *N* = 120	Quasi-random	71.42 (7.18)
Chan, Yu & Li [2011]	Chinese	PT	Cross sectional	China - HIC	Clinical	Peritoneal dialysis patients, *N* = 141	Random	57 (12)
Cheng et al. [2004]	Chinese	PT	Cross-sectional	Hong Kong - HIC	High schools	Adolescents, *N* = 2105	Not stated	14.8 (1.6)
Sing & Wong [2011]	Chinese	PT	Cross sectional	Hong Kong - HIC	College	College students, *N* = 529	Not stated	21.1 (1.77)
Kee-Lee Chou [2000]	Chinese	T & PT	Cross-sectional	Hong Kong - HIC	High schools	Adolescents, *N* = 410	Random	17.5 (0.7)
Liu et al. [2015]	Chinese	PT	Cross sectional	China - HIC	Clinical	Patients with haematological malignancies, *N* = 225	Consecutive	*15–83 [Range]
Wong et al. [2012]	Chinese	PT	Comparative cross-sectional survey	Hong Kong - HIC	1. Clinical 2. Community	1. Caregivers of stroke, Parkinson’s disease, or Alzheimer disease patients, *n* = 55 2. General population, *n* = 61	Not stated	1. caregivers- 72 (6.2) 2. General population- 72 (6.3)
Liu et al. [2015]	Chinese	PT	Cross sectional	China - HIC	University	University students, *N* = 722		19.68 (1.12)
Yeung et al. [2013]	Chinese	PT	Quasi-experimental	USA-HIC	Clinical	Chinese Americans, *N* = 14	Convenience	53 (14)
Yu Ling et al. [2015]	Chinese	PT	Cross sectional	China - HIC	High schools	Adolescents, *N* = 1654	Random	15.85 (1.02)
Hannan et al. [2016]	Creole	T & PT	Longitudinal	USA-HIC	University	Haitian post-partum mothers, *N* = 85	Convenience	45.8 (11.1)
Denis et al. [2015]	French	T & PT	Cross-sectional	France-HIC	Clinical	Post-partum mothers, *N* = 148	Not stated	30.5 (5.1)
Hamza et al. [2012]	Hausa	T	Mixed methods	Nigeria -LMIC	Clinical	Patients with stroke, *N* = 10	Random	51.5 (not provided)
Mohammad et al. [2015]	Hausa	PT	Cross-sectional	Nigeria - LMIC	Clinical	Patients with stroke, *N* = 140	Consecutive	58.8 (13.2)
Vincent-Onabajo et al. [2015]	Hausa	PT	Cross-sectional	Nigeria - LMIC	Clinical	Patients with stroke, *N* = 100	Consecutive	51.4 (13.5)
Park et al. [2011]	Korean	T & PT	Cross-sectional	Korea-HIC	Clinical	Women with diabetes, *N* = 123	Convenience	53.4 (5.9)
Nakigudde et al. (2009)	Luganda	T & PT	Cross-sectional	Uganda- LIC	Clinical	Post-partum mothers, *N* = 240	Systematic	26 (5.7)
Ng* et al. [2010]	Malay	T & PT	Longitudinal	Malaysia-UMIC	Clinical	University students, *N* = 237	Not stated	*19–25 [range]
Ng* et al. [2015]	Malay	PT	Prospective cohort	Malaysia-UMIC	Clinical	Female patients with breast cancer, *N* = 221	Not stated	551 (11.5)
Razali & Yusoff [2014]	Malay	PT	Cross-sectional	Malaysia-UMIC	Clinical	Patients with Schizophrenia, *N* = 70	Universal	33 (9)
Roohafza et al. [2016]	Persian	PT	Cross sectional	Iran-UMIC	Clinical	Patients with irritable bowel syndrome, *N* = 4763	Not stated	Not stated
Bagherian-Sararoudi, et al. [2013]	Persian	T & PT	Longitudinal	Iran-UMIC	Clinical	1.Myocardial patients, *n* = 176; 2. Healthy participants, *n* = 71: *N* = 247	Not stated	1. 56 (9.8)
Ghorbani et al. [2005]	Persian	PT	Cross sectional	Iran-UMIC	Clinical	Parents of pre-term & full-term infants, *N* = 164	Multi-stage sampling	1. pre-term - 27.6 (6.25) 2. full term-28.22 (4.54)
Adamczyk & DiTommaso [2014]	Polish	PT	Cross-sectional	Poland - HIC	University	Young adults, *N* = 417	Not stated	21.14 (2.05)
Adamczyk &Segrin[2015]	Polish	PT	Cross-sectional	Poland - HIC	University	Young adults, *N* = 553	Not stated	23.42 (3.27)
Adamczyk [2013]	Polish	T & PT	Longitudinal	Poland - HIC	University	University students, *N* = 418	Convenience	21.1 (2.1)
Adamczyk & Segrin[2015]	Polish	PT	Cross-sectional	Poland - HIC	University	Young adults, *N* = 553	Not stated	23.42 (3.27)
Martins et al. [2011]	Portuguese	PT	Cross-sectional	Portugal- HIC	Clinical & Online	Adults attempting to get pregnant, *N* = 312	Convenience	32.01 (4.65)
Martins et al. [2012]	Portuguese	T & PT	Longitudinal	Portugal- HIC	Clinical & Online	Adults attempting to get pregnant, *N* = 589	Convenience	33.8 (5.2)
Martins et al. [2014]	Portuguese	PT	Cross-sectional	Portugal- HIC	Clinical & Online	Adults attempting to get pregnant, *N* = 426	Convenience	1.Men- 34.3(6.2) 2. Women-32.3 (4.9)
Guan et al. [2015]	Simplified Chinese	T & PT	Longitudinal	China - HIC	University	University students, *N* = 202	Convenience	21.9 (2.0)
Cobb & Xie [2015]	Spanish	PT	Cross-sectional	USA-HIC	Community	Hispanic immigrants, *N* = 122	Not stated	33.7 (8.2)
Guillén et al. [2015]	Spanish	PT	Cross-sectional	Spain - HIC	Community	Female intimate partner violence victims, *N* = 136	Convenience	31.67 (SD not stated)
Ramos et al. [2016]	Spanish	T & PT	Cross-sectional	Spain - HIC	Community	Retirees, *N* = 991	Convenience	62.7 (5.89)
Rey et al. [2016]	Spanish	PT	Cross-sectional	Spain - HIC	Community	Adults, *N* = 613	Not stated	34.36 (11.18)
Trujols et al. [2014]	Spanish	PT	Cross-sectional	Spain - HIC	Clinical	Patients with depression, *N* = 173	Consecutive	50.2 (14.9)
Ekbäck et al. [2013]	Swedish	T & PT	Cross-sectional	Sweden -HIC	Clinical	1. Patients with Hirsutism, *n* = 127 2. Nursing students, *n* = 154	Not stated	1. Patients with, Hirsutism, 32.0 (10.3) 2. Nursing students, 27.3 (7.8)
Ekbäck et al. [2014]	Swedish	PT	Comparative, cross-sectional	Sweden -HIC	Clinical	1.Patients with Hirsutism, *n* = 127 2. Normative sample, *n* = 1115	Not stated	1. Patients with Hirsutism 32.0 (10.2) 2. Normative sample, 32.7 (7.9)
Guan et al. [2013]	Tamil	T & PT	Cross-sectional	Malaysia-UMIC	University	University students, *N* = 94	Not stated	38.3 (17.9)
Ross et al. [2011]	Thai	PT	Cross-sectional, correlational	Thailand-UMIC	Clinical	Postpartum, HIV-positive women, *N* = 85	Convenience	26.8 (5.64)
Wongpakaran [2011]	Thai	T & PT	Cross-sectional	Thailand-UMIC	University	1. Medical students, *n* = 310 2.Patients with major depressive disorder, *n* = 152 [*N* = 462]	Convenience	1. Medical students, *n* = 19.16 (1.02) 2. Patients with major depressive disorder, 41.23 (12.30)
Wongpakaran [2012]	Thai	T & PT	Cross sectional	Thailand-UMIC	University	Medical students, *N* = 486	Not stated	19.01 (0.90)
Eker & Arkar [1995]	Turkish	T & PT	Cross-sectional	Turkey - UMIC	University & Clinical	1. University students, *n* = 146 2. Patients with renal problems, *n* = 50	Not stated	1. University students,20.34 (1.55) 2. Patients with renal problems,37.18 (12.8)
Ersoy & Varan [2007]	Turkish	PT	Cross-sectional	Turkey - UMIC	Clinical	Patients with psychiatric disorders, *N* = 203	Convenience	33.79 (11.77)
Eker, Arkar & Yaldiz [2000]	Turkish	PT	Cross-sectional	Turkey - UMIC	Clinical	1. Psychiatry patients, *n* = 50 2. Surgery patients, *n* = 50 3. Normative sample, *n* = 50	Convenience	1. Psychiatry patients, 36(13) 2. Surgery patients, 36(13) 3. Normative sample, 35(11)
Kuscu et al. [2009]	Turkish	PT	Cross-sectional	Turkey - UMIC	Clinical	Caregivers of adult cancer patients, *N* = 51	Convenience	Not stated
Duru [2007]	Turkish	PT	Cross-sectional	Turkey - UMIC	University	Students, *N* = 340	Not stated	18.83 (1.35)
Basol [2008]	Turkish	PT	Cross-sectional	Turkey - UMIC	Schools	Administrators, *N* = 433	Not stated	Not stated
Akhtar et al. [2010]	Urdu	PT	Longitudinal	Pakistan- LMIC	Community	Antenatal women, *N* = 325	Not stated	27 (5)
Saleem et al. [2013]	Urdu	PT	Cross sectional	Pakistan- LMIC	Clinical	Drug addicts, *N* = 70	Not stated	32.21 (8.30)
Khan et al. [2015]	Urdu	PT	Cross sectional	Pakistan- LMIC	Community	Pregnant women, *N* = 349	Cluster	< 19–30+ [Range]
Qadir et al. [2013]	Urdu	PT	Cross sectional	Pakistan- LMIC	Community	Married women, *N* = 277	Convenience	36.7 (9.96)
Naveed & Naz [2015]	Urdu	PT	Cross sectional	Pakistan- LMIC	Clinical	Women with postpartum depression, *N* = 100	Not stated	27.31 (5.20)

T- translation: *PT* psychometric testing, *LIC* lower income country, *LMIC* lower-middle income country, *UMIC* upper middle-income country, *HIC* high income country <World Bank Classification system>

##### Description of adaptations

**Fig. 1:**
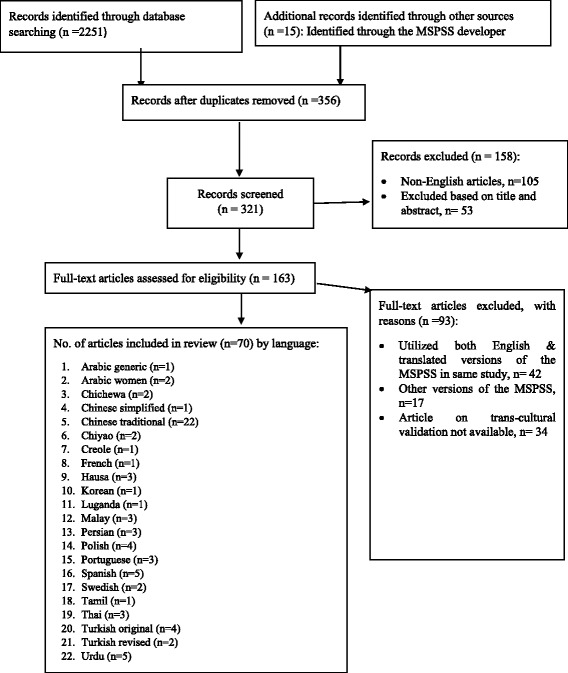
Flowchart of article search and selection process: We identified 2251 articles, of which 356 were duplicates. After applying the selection criterion, 70 articles were analysed for the present review

For seven of the translations, the response options were reduced from the original seven -point Likert scale to a five (*n* = 4), three (*n* = 1), four (*n* = 1) and six-point (*n* = 1) scale. Some of the original terms on the MSPSS were modified/changed in four of the studies i.e. the term special person/significant other was changed to “husband” or “spouse”. The MSPSS was self-administered in most studies [54.5%, *n* = 12]. Depression, general psychological well-being, social networks and anxiety were the most commonly measured secondary outcome measures Fig. 1 and Table 6.

**Table 6:** Adaptations to the MSPSS and outcome measures per study

Language (s)	esponse options	Modification	Mode of administration	Statistical analyses	Secondary outcome measures		
					Measure 1	Measure 2	Measure 3
Arabic	3	Special person changed to Husband	Interviewer administered	CFA	Seeking Social Support, Problem Solving, Blaming Self and Avoidance Scales - Revised Ways of Coping Checklist (RWCCL)	Emotional distress- Profile of Mood States (POMS)	Daily Hassles Scale (DHS)
Arabic	7	None	Not stated	EFA	Emotion Regulation Questionnaire (Arabic-ERQ)	Satisfaction with Life Scale (Arabic-SWLS)	
Chichewa (*n* = 269) & Chiyao (*n* = 314)	5	Questions changed to second person pronouns	self-administered & interviewer administered	1. EFA 2. CFA	Depression – Self Reporting Questionnaire (SRQ)	Depression – Edinburgh Postnatal Depression Scale (EPDS)	Depression – Structured Clinical Interview for DSM-IV (SCID)
Creole	7	none	Self-administered	Parametric & non-parametric tests	Perceived Adequacy of Resource Scale (PARS)		
French	7	None	Not stated	CFA	Depression – Edinburgh Postnatal Depression Scale (EPDS)		
Hausa	7	Terms changed to culturally acceptable equivalents	Interviewer administered	Qualitative			
Hausa	7	None	Not stated	CFA			
Hausa	7	None	Not stated	Regression	Disability- modified Rankin Scale		
Simplified Chinese	4	Not clearly stated	Self-administered	EFA	Psychological symptomatology – GHQ 30	Social networks- Lubben Social Network Scale	
Simplified Chinese	5	None	Self-administered	CFA			
Korean	7	Significant other replaced by “spouse/partner”	Not stated	EFA	Perceived social support- Personal Resource Questionnaire −2 (PRQ)	Self-efficacy- Self -efficacy for Diabetes Scale	Depression- Center for Epidemiological Studies Depression Scale (CEDS)
Luganda	5	use of facial cues	Interviewer administered	EFA			
Malay	7	Not clearly stated	Self-administered	EFA	Social support - Medical Outcome Survey (MOS)	Depression- BDI	Mental health- General Health Questionnaire
Persian	7	Not clearly stated	Not stated	EFA			
Polish	7	None	Self-administered	1. EFA 2. CFA	Loneliness- The Social and Emotional Loneliness Scale for Adults-Short Form (SELSA-S)	The State-Trait Anxiety Inventory (STAI)	Satisfaction with Life Scale (SWLS)
Portuguese	6	None	Self-administered	CFA	Depression – Beck Depression Inventory	Fertility- Fertility Problem Inventory	
Simplified Chinese	7	None	Self-administered	EFA	Social support - Medical Outcome Survey (MOS)	Depression - BDI	Mental health- General Health Questionnaire
Spanish	7	None	Self-administered	EFA	None	None	none
Swedish	7	None	Self-administered	EFA			
Tamil	7	None	Self-administered	EFA	Social support - Medical Outcome Survey (MOS)	Depression – Beck Depression Inventory	Mental health- General Health Questionnaire
Thai	7	None	Self-administered	1.EFA 2.CFA	Anxiety- The Sate Trait Anxiety Inventory (STAI)	Self-esteem-The Roseburg Self-Esteem Scale (RSES)	Depression- Thai Depression Scale (TDS)
Traditional Chinese	7	Not clearly stated	Interviewer administered	1. EFA 2. CFA			
Turkish	7	None	Not stated	EFA	Depression – Beck Depression Inventory	The State-Trait Anxiety Inventory (STAI)	
Urdu	7	None	Interviewer administered	EFA	Mental distress- Self Report Questionnaire (SRQ-20)	Depression - EPDS	

*EFA* exploratory factor analysis, *CFA* confirmatory factor analysis

### Results of individual studies

#### Arabic

Two variants of Arabic translations were retrieved [27, 35].

#### Arabic generic version

The Arabic generic version was described in one study [27]. The methodology for this cross-cultural validation study was poor, as scanty details were provided for the adaptation process. The evidence for structural validity was poor as only exploratory factor analysis (EFA) was performed. There was limited evidence for internal consistency (IC) as the handling of missing responses was not reported. There was unknown evidence for construct validity as no specific hypotheses were formulated.

#### Arabic version for women (MSPSS-AW)

Two studies on the MSPSS-AW were available [35, 63]. The methodology for the cross-cultural validation was poor. There was no description of; translators’ expertise, whether the translations were done independently, the number of forward and backward translations performed and the reconciliation process. This version was not reviewed by a committee and was not pretested. There was moderate evidence for IC, the handling of missing responses was not reported. There was fair evidence for structural validity, although confirmatory factor analysis (CFA was performed, the number of missing responses was not documented. There was limited evidence of construct validity as no specific hypotheses were formulated and the psychometrics of the comparator instruments were not adequately described.

### Chichewa and Chiyao

Two studies were available [26, 36]. The methodology for the cross-cultural validation was fair as only one forward and one backward translations were performed. Further, the expertise of the translators, pre-test sample and the reconciliation of the forward translation were poorly described. There was strong evidence for structural validity and IC. Both EFA and CFA were applied for structural validity evaluation. There was limited evidence for construct validity, no specific hypotheses were formulated.

### Chinese

Two versions in simplified and traditional Chinese versions were retrieved [12, 17].

#### Traditional Chinese

Twenty-one studies, applying the traditional Chinese version of the MSPSS, were available [17, [64–84]. The methodology for the cross-cultural validation was poor. Solitary forward and backward translations were performed; the translators’ expertise were not outlined and it was not clear whether the translations were done independently and if the tool was pretested in the target population. Two studies tested structural validity of the MSPSS-C in different populations [17, 64]. There was poor evidence for structural validity as only EFA was performed. There was conflicting evidence for IC. The methodological quality of one of the studies was questionable [17] with the second study yielding a Cronbach’s alpha< 0.70 despite fair methodological quality [65]. There was limited evidence for reliability as the test conditions and the stability of the re-test sample were not clearly outlined. There was moderate evidence for construct validity, no specific hypotheses were formulated. The evidence for criterion validity was unknown as the psychometrics of the “purported” gold standard measure was questionable.

#### Simplified Chinese

Only one study was available [12]. The methodology for the cross-cultural validation was poor. The credentials of the translators were not clearly described; it was not clear if the translations were done independently and the tool was not pretested. The evidence for criterion validity, construct validity and reliability was indeterminate. No information was provided on the psychometric robustness of comparison outcome measures, the time for the re-test was inappropriate and no specific hypotheses were formulated.

### Creole

Only one study was available [6]. The tool was poorly translated as; there were no multiple translations, the tool was not pre-tested and factorial analysis was not done. The evidence for IC was indeterminate as the subscales unique ICs were not computed and handling of missing responses was not documented. There was indeterminate evidence for reliability as the conditions for the administrations were not clearly stated and the evidence for no systematic change in the outcomes was not provided.

### French

Only one study was available [9]. The cross-cultural translation and adaptation process was poor. The tool was not pre-tested and only solitary forward-backward translations were performed. There was limited evidence for structural validity, IC, reliability and construct validity. The methodologies applied were of fair quality, the handling of missing response was not reported, the conditions for the test-retest were not clearly outlined and no specific hypotheses were formulated respectively.

### Hausa

Three studies were available [7, 14, 85]. The methodology for the cross-cultural validation was good. A solitary backward translation was done and reconciliation process was poorly described. There was strong evidence for structural validity and IC. There was limited evidence for test-retest reliability and construct validity. It was not clear if administrations were independent, if patients were stable in between administrations and the MSPSS was re-administered after a week against the recommended 2 weeks [57, 86]. Further, the handling of missing responses was not reported and no specific hypotheses were formulated.

### Korean

One study was available [87]. The methodology for the cross-cultural validation was poor. There was a scanty description of the expertise of the translators and whether the translations were done independently. It was not clear if the tool was pretested in the target population and solitary forward and backward translations were performed. There was poor evidence for structural validity as only EFA was performed. There was strong evidence for IC as the methodology was of excellent quality. There was limited evidence for construct validity, no specific hypotheses were formulated.

### Luganda

One study was available [13]. The methodology for the cross-cultural validation was fair. A solitary forward translation was performed; the characteristics of the pre-test sample were not clearly described and details of the reconciliation of the original and forward translation were scanty. There was unknown evidence for structural validity as only EFA was performed. Further, an inappropriate rotation method (orthogonal rotation) was applied for EFA. There was limited evidence of the IC, the handling of missing responses was not documented.

### Malay

Three studies were available [11, 88, 89]. The methodology for the cross-cultural validation was poor. There was a scanty description of; the expertise of the translators, whether the translations were done independently, the reconciliation process, and the tool was not pretested in the target population. There was poor evidence for structural validity as only EFA was performed. There was indeterminate evidence for IC, the handling of missing responses was not documented. There was unknown evidence for construct validity; no specific hypotheses were formulated with poor/no description of the psychometrics of the comparator instruments. There was no report of test-retest reliability; the stability of the respondents was not clearly outlined; the tool was re-administered after a week and there was a disparity in administration conditions as the items were reshuffled for the retest. There was unknown evidence for criterion validity, the psychometrics for the purported “gold standard” outcome measure was not provided.

### Persian

Three studies were available [18, 90, 91]. The methodology for the cross-cultural validation was poor. The expertise of translators, handling of missing responses and reconciliation process was poorly described. Further, solitary forward and backward translations were performed and the tool was not pre-tested. There was poor evidence for structural validity as only EFA was performed. There was limited evidence for IC and reliability as the methodologies were of fair quality. Only 71 participants were recruited for test re-test reliability and the conditions and stability for the re-test sample were not clearly stared. There was unknown evidence for construct validity as no specific hypotheses were formulated and no the psychometrics of the comparator instruments were not provided.

### Polish

Four studies were available [92–95]. The methodology for the cross-cultural validation was good. The reconciliation of the translations was poorly described and the tool was not reviewed by a committee. There was strong evidence for IC and structural validity. There was moderate, negative evidence for construct validity as no specific hypotheses were formulated.

### Portuguese

Three studies were available [31, 96, 97]. The methodology for the cross-cultural validation was fair. The expertise of the translators was not stated, if was not clear if translations were done independently, only solitary forward and backward translations were done and the tool was not reviewed by a committee. There was excellent evidence for both structural validity and IC. There was unknown evidence for test-retest reliability; a sub-optimal sample (*n* = 52) was utilized, the stability of the participants and the conditions for the re-test were not stated. There was limited evidence for construct validity as no specific hypotheses were formulated.

### Spanish

Five studies were available [34, 98–101]. The methodology for the cross-cultural validation was poor. The expertise of the translators was not stated; only solitary forward and backward translations were done and the tool was not pre-tested. There was conflicting evidence for structural validity as the cited studies were of both poor and fair quality. For instance, for one of the studies, EFA contrary to the CFA reported was done and authors performed varimax (orthogonal) rotation [101]. There was limited evidence for IC as the handling of missing responses was not recorded. There was moderate evidence for construct validity as no specific hypotheses were formulated.

### Swedish

Two studies were available [3, 102]. The methodology for the cross-cultural validation was poor. A solitary backward translation was performed; the handling of missing responses was not reported and the credentials of the translators were not clearly described. Evidence for structural validity was poor, only EFA was performed and an inappropriate rotation method (orthogonal varimax) was utilized. There was moderate evidence for IC, the handling of missing responses was not described. Evidence for reliability was limited as a sub-optimal sample size (*n* = 44) was used for the retest and the conditions of the re-test administration were not clearly described. There was limited evidence for construct validity as no specific hypotheses were formulated.

### Tamil

Only one study was available [103]. The methodology for the cross-cultural validation poor. There was scanty description of: the expertise of the translators was, if forward translations were done independently, the reconciliation process, whether the tool was not pre-tested in the target population and the profile of the pre-test sample. There was limited evidence for IC, a sub-optimal sample size (*N* = 94) was recruited and handling of missing responses was not reported. There was unknown evidence for structural validity, construct validity and criterion validity. Only EFA was performed, no specific hypotheses were formulated and the psychometrics of the purported “gold standard” were not provided.

### Thai

Three studies were available [28, 43, 44]. The methodology for the cross-cultural validation was poor. The forward translators did not work independently; only solitary forward and backward translations were performed and scanty details were provided for the reconciliation process and the pre-test sample profile. There was moderate evidence for structural validity and IC. Both EFA and CFA were performed, however, the percentage of missing responses was not stated. There was unknown evidence for construct validity as the no specific hypothesis were formulated. There was limited evidence for test-retest reliability, a suboptimal sample (*N* = 72) was utilized, the conditions and stability of patients for the re-test were not clearly outlined.

### Turkish

Two versions of the Turkish translations were available i.e. the original Turkish version [39, 41] and the revised Turkish version [29, 104].

#### Original Turkish version

Four studies were available [39–42]. The methodology for the cross-cultural validation was poor. A solitary forward translation was performed; it is not clear if the forward translators worked independently and the tool was not pre-tested. There was poor evidence for structural validity as CFA was not performed. There was moderate evidence for IC, the handling of missing responses was not described. There was unknown evidence for construct validity as no specific hypotheses were formulated and one of the studies was of poor methodological quality [41].

#### Revised Turkish version

Two studies were available [29, 104]. There was moderate evidence for structural validity as the handling of missing values was not described. Evidence was; conflicting for IC and unknown for reliability and construct validity. A sub-optimal sample was utilized; the test conditions and stability of the participants was neither described and no specific hypotheses were formulated.

### Urdu

Five studies were available [45–49]. The methodology for the cross-cultural validation was poor. The following were not stated; the expertise of the translators, if translations were done independently, the number of forward and backward translations. Further, the tool was not reviewed by a committee and was not pretested in the target population. There was limited evidence for structural validity [47, 49]. One of the studies was of poor quality, only EFA was performed [49]. For the second study, although CFA was performed, the handling of missing responses was not documented [47]. There was indeterminate evidence for IC as the methodology was of poor quality. There was moderate evidence for construct validity, no specific hypotheses were formulated.

## Discussion

### Settings

The MSPSS has been translated across a range of settings and populations.

### Translation quality

Trans-cultural adaptation, translation and validation aim to succinctly capture the meaning of a latent construct in another population. As such, a rigorous translation process is essential [25, 52]. None of the studies included in this review were translated using robust methodologies, with 16 of the 22 studies being of poor methodological quality in accordance with the COSMIN criteria [56]. The lack of quality of the translations affects the generalizability and comparability of the study findings. For example, if the MSPSS is applied in a large multi-national trial, there is risk of misleading results if one of the translations was poorly conducted. The findings could have negative implications on policy formulation, over−/under estimation of an intervention effect size amongst other.

The lack of detailed descriptions of both language and construct expertise of the translators, whether the translations were done independently and reconciliation of the translations compromised the methodological rigor of most of the retrieved studies. Furthermore, the absence of a panel of experts review process for content and face validation, as was the case in 13 of the 21 included studies, could have jeopardised the ability to produce a culturally acceptable translation [25, 52]. Ideally, the panel should consist of experts with diverse professional backgrounds to ensure the attainment of semantic, idiomatic, and conceptual equivalence [25]. For instance, given the differences in cultures, the interpretation of the term “special person” can vary from setting to setting. It is argued that respondents from collectivistic cultures may not distinguish between family and a significant other as sources of SS [47, 49]. For example, in Turkey, when the term “special person” was changed to “husband” following a panel of experts’ review, the resultant/revised translation yielded a three-factor structure as opposed to the earlier two-factor structure [29, 104]. This illustrates that a more rigorous reconciliation and adaptation can yield a more reliable factor structure. Lastly, pretesting/cognitive debriefing of the translated and adapted tool is essential before the tool can be applied to a larger population [25, 52, 56]. This should be done in the target population as translation is an integrated and iterative process and requires input from “experts” and the “target users” of the PROM [52]. Unfortunately, only five of the 23 translations described this process in detail, including description of sample selection, hence this could also be a source of methodological limitation for the retrieved studies.

### Structural validity

Structural/factorial validity is defined as the extent to which scores on an outcome measure adequately reflect the dimensions/structure of the construct to be measured [41]. Factorial validity can be envisaged as the ‘backbone’ for the statistical evidence of the validity or lack thereof of a translated tool. Ideally for translated outcome measures, both EFA and CFA should be performed to test factorial validity [3, 31]. EFA is a technique used to explore/discover the number of factors a tool possesses [105–107]. The original MSPSS has a three-factor structure, it is essential to test if this is the same for the translated versions as SS is a multidimensional, subjective construct which is dependent on socio-cultural contextual factors [1, 3, 10–12, 17, 18, 108]. To this end, it is acceptable to obtain a one- or two-factor structured translation if the translation method is adequately robust. However, EFA alone is inadequate, as was the case in most of the retrieved translations; therefore, CFA ought to also have been performed [57, 86]. CFA is an advanced structural equation modelling statistical technique which combines the concepts of EFA, correlation and multiple regression [109, 110]. It provides evidence as to whether the translated versions replicated the original three-factor structure as postulated by the developers of the MSPSS. Unfortunately, a minority of the studies [9/23] performed CFA with only four studies [4/23] performing both EFA and CFA which is a major shortcoming for the level of evidence for structural validity. Further, in some instances, some authors/studies refer to EFA as CFA [28, 43, 44, 103] and this again yields inaccurate conclusions. In instances were only EFA was performed, some authors utilized an inappropriate rotational method i.e. orthogonal instead of oblique rotation [3, 13, 102]. Orthogonal rotation is used when the factors are hypothesized to be unrelated [105, 107, 111], which is not the case for the MSPSS as the domains are stipulated to be correlated [15, 21, 22]. For studies which performed CFA, only three adequately described the goodness of fitness (GOF) indices. These are important as they provide concrete evidence to the degree to which the data/translation fits into the original factor model [109, 110, 112]. Furthermore, given that the MSPSS can yield one-, two- or three- factors, all the three models should be tested using CFA before a decision on the degree of fit can be made. None of the studies which performed both EFA and CFA included this analysis, hence this could be envisaged as a potential source of reporting bias. Replication of the original factorial structure is not necessarily a benchmark for an accurate translation process [25]. For instance, authors may be tempted not to report the results of a two-factor model if the degree of fit is much better than for a three-factor model. Provision of multiple GOF indices for all the three models should be a “standard” reporting practise as it provides the potential readership with all the essential information for them to critique the methodological quality and subsequent conclusions in keeping with the evidence supplied [110].

### Reliability

Most of the translated tools displayed adequate evidence for IC as most attained a Cronbach’s alpha of at least 0.70. However, given the limitations in the structural validity testing and lack of rigour in the translation process, the results for IC may need to be interpreted with caution. This is because a tool can be reliable, yet not valid [25, 113]. To illustrate this, if only EFA is performed, the factorial validity will be poor, however, the tool can still yield a high alpha statistic. In that instance the reliability findings can be deemed as “misleading” [114]. The validity of the alpha scores is also dependent on the homogeneity/unidimensionality of a test and this can be established through factor analysis [56]. Therefore, if CFA (the preferred unidimensionality test for translated tools) is not performed, the IC for that test will/may not be valid [56, 114, 115]. Additionally, the longitudinal validity (test-retest) also gives further evidence of the stability of an outcome measure over time [56]. Only four studies reported the stability of the translated versions which is another potential limitation. Given the potential limitation in relying solely on the IC as an indicator of reliability, other indices such as the alternative forms, split-half and test-retest reliability are recommended for concrete evidence of reliability of outcome measures [113, 115]. More so, it is argued that despite its wide usage and popularity, the Cronbach alpha is least desirable index for reliability estimation [115].

### Construct validity

Construct validity refers to the extent to which scores on an instrument relate to other measures in a manner that is consistent with theoretically derived hypotheses concerning the concepts that are being measured [54]. Depression, anxiety, self-esteem and general mental well-being were the most commonly reported outcomes against which SS scores were compared. To prevent report bias, the developers of the COSMIN checklist recommend that authors should formulate specific hypothesis before data collection [54, 57, 86]. None of the studies specified the expected magnitude of correlations with only three studies formulating specific hypotheses. Further, there is need for authors to describe in detail the comparator instruments as well as demonstrating their reliability and validity in the study population as failure to do so affects the both internal and external validity [86]. For example, some of the translations [eight out of twenty-three] did not report the psychometrics of the comparator instruments. In other instances, the authors refer to the psychometrics of the comparator from another population which again is questionable [45–49]. Failure to demonstrate the psychometric robustness of the comparator instruments would thus affect the construct validity of the translated versions of the MSPSS.

### Criterion validity

Criterion validity is defined as the extent to which scores on an outcome measure perform against an established gold standard [58]. Given that SS is a latent variable [3, 11], it is difficult to establish a gold standard against which the MSPSS can be assessed against. Nevertheless, for the three studies which evaluated criterion validity, the psychometrics of the purported gold standard were either questionable or were not well described. Therefore, there was poor evidence for criterion validity.

### Limitations

The use of the COSMIN checklist for the evaluation of the methodological quality may have been a potential limitation. This is because the checklist came into effect in 2011 and some of the translations had been performed prior to its’ publication. The stringent nature of the checklist has also been reported in almost similar systematic reviews [24, 116]. For example, in the assessment of IC and factorial validity, if the handling of missing responses is not reported, the domain(s) are rated as fair quality despite the rest of the ratings being of excellent quality. Inconsistencies within the COSMIN checklist may also be viewed as a potential source of limitation. For example, in evaluating the structural validity of translated tools, if CFA is not performed, item 6 for the structural validity/Box E is rated as good and the same is rated as poor for item 14 under Box G/cross-cultural validity. As the COSMIN guidelines are currently under review, it is hoped the revised guidelines will further harmonize the terminology utilized in the methodological and further increase the checklist validity in rating methodological quality of the translation and adaptation of PROMs. Additionally, we could not evaluate fifteen language versions of the MSPSS which were published in other than English language and this could have introduced language bias for the present review.

## Conclusions

We identified 22 translated versions of the MSPSS. The psychometric properties which were most often reported included internal consistency, test-retest reliability, structural validity and construct validity. Many of the tools did not follow a rigorous translation process and there was poor evidence for structural validity. The advent of EBP and increased usage of PROMs requires quality translations to ensure reliable and valid outcome measures. The retrieved MSPSS translations therefore need to be utilized with precautions. There is also need to assess other psychometric properties such as responsiveness, measurement error and establishment of cut-off values to increase the clinical utility and psychometric robustness of the translated versions of the MSPSS. We also recommend the development of a standardized protocol for the translation and adaptation of the MSPSS. Future translation studies should utilize the backward-forward translation method with special emphasis on the use of multiple translators, reconciliation of translations, panel of expert assessment and both EFA and CFA should be performed for factorial analysis.

## Additional files

Additional file 1: Multidimensional Scale of Perceived Social Support [MSPSS]. (DOC 35 kb)

Additional file 2: Populated PRISMA 2009 Checklist. (DOC 63 kb)

## Abbreviations

CFA: Confirmatory factor analysis
COSMIN: COnsensus-based Standards for the selection of health status Measurement Instruments
EBP: Evidence based practise
EFA: Exploratory factor analysis
HIC: High-income country
IC: Internal consistency
LIC: Low income country
LMIC: Lower-middle income country
MIC: Middle income country
MSPSS: Multidimensional Scale of Perceived Social Support
PROMs: Patient-reported outcome measures
SS: Social support
UMIC: Upper middle income country
