# Supplementary material for: A systematic review of the psychometric properties of the cross-cultural translations and adaptations of the Multidimensional Perceived Social Support Scale (MSPSS)
Source: Health Qual Life Outcomes. 2018 May 2;16:80. doi: 10.1186/s12955-018-0912-0 (PMC5930820; doi:10.1186/s12955-018-0912-0)
Supplement: Supplementary file 1 — Multidimensional Scale of Perceived Social Support [MSPSS]. (DOC 35 kb) [file 12955_2018_912_MOESM1_ESM.doc]

# ADDITIONAL FILE 1: Multidimensional Scale of Perceived Social Support [MSPSS]

**Instructions:** We are interested in how you feel about the following statements. Read each statement carefully. Indicate how you feel about each statement.

Circle the “1” if you **Very Strongly Disagree**

Circle the “2” if you **Strongly Disagree**

Circle the “3” if you **Mildly Disagree**

Circle the “4” if you are **Neutral**

Circle the “5” if you **Mildly Agree**

Circle the “6” if you **Strongly Agree**

Circle the “7” if you **Very Strongly Agree**

Very Very

Strongly Strongly Mildly Mildly Strongly Strongly

Disagree Disagree Disagree Neutral Agree Agree Agree

1. There is a special person who

is around when I am in need. 1 2 3 4 5 6 7

2. There is a special person with

whom I can share joys and sorrows. 1 2 3 4 5 6 7

3. My family really tries to help me. 1 2 3 4 5 6 7

4. I get the emotional help & support

I need from my family. 1 2 3 4 5 6 7

5. I have a special person who is

a real source of comfort to me. 1 2 3 4 5 6 7

6. My friends really try to help me. 1 2 3 4 5 6 7

7. I can count on my friends when

things go wrong. 1 2 3 4 5 6 7

8. I can talk about my problems with

my family. 1 2 3 4 5 6 7

9. I have friends with whom I can

share my joys and sorrows. 1 2 3 4 5 6 7

10. There is a special person in my

life who cares about my feelings. 1 2 3 4 5 6 7

11. My family is willing to help me

make decisions. 1 2 3 4 5 6 7

12. I can talk about my problems with

my friends. 1 2 3 4 5 6 7
